# Viruses: Friends or foes

**DOI:** 10.1111/1751-7915.13948

**Published:** 2021-10-11

**Authors:** Carmen Michán, Alfredo Michán‐Doña

**Affiliations:** ^1^ Departamento de Bioquímica y Biología Molecular Universidad de Córdoba Campus de Excelencia Internacional Agroalimentario CeiA3 Campus de Rabanales Edificio Severo Ochoa Córdoba E‐14071 Spain; ^2^ Departamento de Medicina Hospital Universitario de Jerez Jerez Spain; ^3^ Biomedical Research and Innovation Institute of Cadiz (INiBICA) Cádiz Spain

## Abstract

Importance of viruses for biotechnological processes.

Biological evolution can be defined as the change of inherited traits over successive generations in populations. Evolution is also related to adaptation, the process by which altered organisms become better suited for survival in specific ecological habitats. Thus, the instability of the environment conditions the mechanism of adaptation, the so‐called r/k strategies. ‘r’ populations are related to unpredictable environments and produce many offspring, thus have more options of mutations, whereas ‘k’ strategists live in more stable niches and have only a few descendants.

A clear example of ‘r’ organisms are viruses. Viruses are the simplest forms of life, just a nucleotide chain that can be naked or wrapped in a bilipid coat. This polynucleotide typically encodes the polymerase needed for its replication that have usually a reduced fidelity, so the virus rate of mutation is high. Error‐prone replication can only occur in organisms with low nucleotide complexity, so that the number of accumulated mutations in a replicative event does not lead to an ‘error catastrophe.’ On the other hand, viruses are obligatory cellular parasites, and this dependence results in a highly hostile environment. Viruses´ polymerase fidelity is fine‐tuned by natural selection to optimize their diversity, but not to generate too many lethal mutations that would cause the extinction of the population. Moreover, recombination is also a main driving force in virus evolution. The final consequence is that viruses have an extraordinary capacity of evolution.

## Number and Types

Viruses dominate all habitats of life and play a primary role in the control of populations. Furthermore, along with humans, they are the most devastating organisms to all types of life. Their abundance is simply stratospheric, so that if we focus on the ocean microbiome it is estimated that there are 10^7^ viruses per ml what sums up to 10^31^ in the global ocean. Virus particles are responsible for the death of 20% of their microbial biomass daily, so that nutrient and energy cycles rely on them (Suttle, 2007; Dávila‐Ramos *et al*., 2019; Dance, 2021; Michán *et al*., 2021). No need to say that the vast majority are unknown.

To date, most of the viruses investigated are related to human pathogenesis but if COVID‐19 pandemic has taught us something, it is the importance of the ‘One Health’ concept. This is not a novel concept since Gaia goddess first appeared in the ancient Greek Mythology, and this idea of considering the Earth as a whole organism has subsequently been widely discussed.

Lately, high‐throughput sequencing has provided hundreds of new viral sequences, and now we face the task of classifying and naming all of those viruses. Actually, hundreds/thousands of environmental genomes and transcriptomes have already been sequenced. Would it be useful to re‐analyse them looking for virus sequences? That takes us to the next problem, are there common virus sequences? The answer is not as far as we know. There are several virus databases that have certainly contributed to new identifications, but viruses do not have a common ancestor and they have arisen at different times of evolution. So, we are probably missing an indeterminate number of new classes with unknown sequences, functions and effects.

## Uses in *Microbial Biotechnology*

Could we use virus particles as instruments or weapons for microbial biotechnological applications? The answer is clearly yes. Virus vectors have been in use for years. Their simplicity provides an easy tool to construct multiple modifications by genetic engineering approaches.

Already in 1950s, the myxoma virus was introduced deliberately in Australia to control the European rabbit overpopulation. Bur, although initially the virus killed almost all the bunnies, the remained animals adapted and acquired genetic immunity over time. Currently, several viruses are being used for the control of pests. Some of them have been genetically engineered and are produced as commercial products, but many others are naturally occurring. Baculovirus is arthropod‐specific large circular double‐stranded DNA commonly used for the control of insect pests in plants due to their very specific target spectrum, thus limiting their side‐effect in other no pathogenic species (Srinivasan *et al*., 2019). Their use is safe and vegetable products are free of blemish and chemicals, which makes them preferred by consumers and provides higher market value.

In the pharmacological field, viral therapies have several advantages, such as high specificity, low drug resistance and low cost. Several vaccines are based on adenoviruses, although their unwanted secondary effects have generated a great controversy lately. Several adverse effects have been linked to adenovirus‐vectored vaccines against SARS‐CoV‐2 or other viruses; most of them were mild, but rare cases of pathologies, such as thrombosis, severe pneumonia, Bell’s palsy, Guillain‐Barré syndrome, gait disturbance, and transverse myelitis, have been reported. We should also bear in mind that the massive and simultaneous current vaccine inoculation is providing a huge amount of new data, including those on the occurrence of these adverse reactions.

One of the most promising applications of viral biotechnology is the use of genetically modified viruses to revert diseases. Phages have an extraordinary potential for treating bacterial infections. Target‐specific viruses to combat multi‐resistant infections have completed several clinical trials that generated almost no adverse events (Fernández *et al*., 2011; Liu *et al*., 2021). Besides infection treatments, oncolytic virotherapy is a very active and promising field of research. Natural and engineered viruses have shown the ability to selectively infect and lyse tumour cells. Furthermore, they also have a second way of action that may contribute to tumour eradication. Tumour cells have weak immunogenicity, mainly due to low levels of cytokines and to the loss of cell ligands that would normally be recognized by innate immune cells. Oncolytic viruses produce a variety of changes in the tumour microenvironment that stimulate antitumor innate immune responses and that increase the success of tumor eradication particularly when oncolytic viruses are combined with radiation or chemical treatments. On the other hand, this innate immune response also dampens virotherapies as it activates the antiviral response of the body (Altomonte and Ebert, 2012; Gao *et al*., 2021; Mealiea and McCart, 2021). Finally, viral particles can be used as couriers of functional genes in cell and gene therapies, both *in vivo* and *ex vivo*. Viral vector‐mediated gene transfer is being tested to counter or replace malfunctioning genes related to urinary tract malformations, metabolism‐related diseases, neuronopathies or haemophilia, among others. They are based basically on five viral vectors: adenovirus, adeno‐associated virus, lentivirus, retrovirus and herpes viruses. Virus fetal gene therapies to amend developmental defects are being trialled in animal models. The development of these therapies has flourished lately due to the discovery and development of the CRISPR‐Cas gene‐editing system. Nevertheless, the contribution of these viruses to our long‐term health is so far largely unknown, e.g., modified phages can alter the intestinal bacteriome and/or phageome or provoke undesirable gene transfer. Additionally, technical limitations and ethical issues need to be addressed.

Viruses have been also shown to be excellent biomarkers for pathogen detection, e.g., faecal pollution in aquatic environments. The use of pathogen levels (including viruses) in wastewater, aka wastewater epidemiology, to monitor infection evolution has been attracting much attention lately. Currently, the levels of SARS‐CoV2 in sewages are being widely used to determine the real infection rate of a certain community/population although there are several limitations (Highlight under progress!).

Could pollution and diseases be tuckered simply by designing viruses that kill certain micro‐organism and/or change the architecture of microbiomes? Our feeling is that this will come true sooner rather than later. We will be able to design viruses that revert genetic alterations or stop bacterial infections. But, could we control the evolution of those ‘artificially designed’ viruses? Could we really ‘domesticate’ them?

## Viruses and humans

The current human population is close to 8 billion. Due to this overpopulation, we are invading ecosystems that were not previously exposed to our species, looking for natural resources and/or food. By doing so, we are increasing the risks of zoonotic transmission of new pathogens, including viral particles. Densely populated cities, together with environmental variations, are the best breeding ground for infection transmission, as recently demonstrated for SARS‐CoV‐2 pandemic (Smith *et al*., 2021).

Viruses are not only responsible for life destruction but also for the creation of genetic novelty. In humans, 8% of our genome are recognisable proviruses and more than 30% are virus‐related retrotransposons. Some of them entered our hominin germline genome millions of years ago. Many have reverse‐transcription activities that could have been gene duplication motors, essentially contributing to our evolution (Mustelin and Ukadike, 2020). This interaction is dynamic and reciprocal, we have shaped them and they have certainly contributed to the actual function of our genome.

In conclusion, viruses can certainly provide useful tools for the control of medical and environmental problems, but we are far from being able to domesticate them. They could be modified and applied for several beneficial purposes, although it is paramount not to overlook their potential risks. Furthermore, in nature, their modulation capacity of the food and energy supplies, and health of cell‐organisms, may situate them as the last resort to prevent overpopulated species. We should assume our responsibility for the fact that the current pandemic has been exacerbated by human demographics and global climate change, which have provoked the overexploitation and abuse of natural resources. We all would like to stay alive and healthy for as long as possible, but viruses may be the last guardians of biology laws limiting our lifespan. What if we all need to die for the future of Gaia?

## Funding Information

No funding information provided.

## Conflict of interest

The authors have no conflict of interest to declare.
